# Comparative Investigation of Chemical Constituents of Kernels, Leaves, Husk, and Bark of *Juglans regia* L., Using HPLC-DAD-ESI-MS/MS Analysis and Evaluation of Their Antioxidant, Antidiabetic, and Anti-Inflammatory Activities

**DOI:** 10.3390/molecules27248989

**Published:** 2022-12-16

**Authors:** Ilhame Bourais, Salma Elmarrkechy, Douae Taha, Bouabid Badaoui, Yassine Mourabit, Najoua Salhi, Mohammed Merae Alshahrani, Ahmed Abdullah Al Awadh, Abdelhakim Bouyahya, Khang Wen Goh, Ching Siang Tan, Souad El Hajjaji, Nadia Dakka, Naima Iba

**Affiliations:** 1 Laboratory of Human Pathologies Biology, Department of Biology, Faculty of Sciences, Genomic Center of Human Pathologies, Mohammed V University in Rabat, Rabat BP 1014, Morocco; 2 Laboratory of Spectroscopy, Molecular Modeling, Materials, Nanomaterials, Water and Environment, Faculty of Sciences, Mohammed V University in Rabat, Rabat BP 1014, Morocco; 3 Laboratoire de Biodiversité, Ecologie et Génome, Faculté des Sciences, Université Mohammed V in Rabat, Rabat BP 1014, Morocco; 4 Laboratory of Pharmacology and Toxicology, Faculty of Medicine and Pharmacy, Mohammed V University, Rabat BP 10100, Morocco; 5 Department of Clinical Laboratory Sciences, Faculty of Applied Medical Sciences, Najran University, 1988, Najran 61441, Saudi Arabia; 6 Faculty of Data Science and Information Technology, INTI International University, Nilai 71800, Malaysia; 7 School of Pharmacy, KPJ Healthcare University College, Nilai 71800, Malaysia

**Keywords:** *Juglans regia* L., plant parts, HPLC-DAD-ESI-MS/MS, phenolic profile, antidiabetic activity, anti-inflammatory, antioxidant

## Abstract

Leaves, husk, kernels, and bark methanolic extracts of *Juglans regia* L. were tested for their in vitro antidiabetic, anti-inflammatory, and antioxidant activities. For these purposes, α-amylase and α-glucosidase were used as the main enzymes to evaluate antidiabetic activities. Moreover, lipoxidase and tyrosinase activities were tested to estimate anti-inflammatory properties. Antioxidant properties of *Juglans regia* L., extracts were determined using three different assays. Leaves extract has an important radical scavenging activity and a-amylase inhibition. Similarly, husk extracts showed high total phenolic content (306.36 ± 4.74 mg gallic acid equivalent/g dry extract) with an important α-amylase inhibition (IC50 = 75.42 ± 0.99 µg/mL). Kernels exhibit significant tyrosinase (IC50 = 51.38 ± 0.81 µg/mL) correlated with antioxidant activities (*p* < 0.05). Husk and bark extracts also showed strong anti-lipoxidase activities with IC50 equal to 29.48 ± 0.28 and 28.58 ± 0.35 µg/mL, respectively. HPLC-DAD-ESI-MS/MS analysis highlights the phenolic profile of methanolic extracts of *Juglans regia* L. plant parts. The identified polyphenols were known for their antioxidant, antidiabetic (dicaffeoyl-quinic acid glycoside in kernels), and anti-inflammatory (3,4-dihydroxybenzoic acid in leaves) activities. Further investigations are needed to determine molecular mechanisms involved in these effects as well as to study the properties of the main identified compounds.

## 1. Introduction

Oxidative stress and inflammation have been considered important factors linked to diabetes mellitus, chronic pulmonary, cardiovascular, and Alzheimer’s diseases, in addition to the physiopathology of cancer. The oxidative stress is known by the overproduction of ROS (reactive oxygen species) leading to the oxidation of macromolecules, notably lipids, proteins, and nucleic acids, which causes the impairment of cellular functions and apoptosis [1,2,3]. Inflammation is a protective process that involves an arsenal of enzymatic reactions and cellular activation. The prolongation of this process causes the loss of cell imbalance and biological system damage [4]. Thus, for better-multifaceted disease management, it is necessary to develop new drugs implying antioxidant and anti-inflammatory activities. In this context, medicinal plants and derivatives have been widely used for therapeutic purposes to heal and protect against several illnesses. Bioactive compounds extracted from natural products can offer specific properties that could act on specific targets and exhibit several biological properties for treating particular diseases [5,6,7]. The use of medicinal plants has increased in recent years. Therefore, screening biological activities remains an important step in therapeutic virtues research. In vitro assays, including enzyme inhibition assays, are commonly used for biological properties, bioactive natural compounds screening, and also for drug synthesis [8,9].

*J. regia* is a well-known worldwide species of the *Juglans* genus belonging to the family of *Junglandacae*. Fruits are consumed for their nutritional value phytotherapy [10,11,12] and have been widely used in folk medicine, especially for diabetes and inflammatory diseases [13] like eczema [14] and gout disease [15], particularly in Morocco [16], Turkey [17], Iran [18], Italy and Romania [19]. Leaves are used to treat digestive disorders like stomatitis, oral ulcer, and diarrhea [14,20,21,22]. Elsewhere, bark’s preparation, also called souak, is used for teeth hygiene and to treat bucco-dentaire sphere problems such as gum diseases, halitosis, and dental stain due to its depurative and antiseptic properties [10,23,24,25].

The literature reported several biological activities exhibited by *J. regia* extracts, including anti-inflammatory and antioxidant activities [26,27,28]. Each part of the plant showed different properties with regard to the cultivars [29], extraction method ([30], and geographic conditions [31]. Moreover, the *J. regia* phytochemical composition varied according to those parameters but the main molecules found are polyphenols, including phenolic acids (gallic acid, vanillic acid, syringic acid, ellagic acid, caffeic acid, ferulic acid, sinapic acid, chlorogenic acid), flavonoids, and tannins [32].

*J. regia* extracts act on different inflammatory mediators; husk extracts can inhibit the nitric oxide (NO) production in macrophages [28], kernel extracts inhibit the activation and the expression of vascular cell adhesion molecule-1 (VCAM-1) and intercellular adhesion molecule-1 (ICAM-1) [33] and are involved in the diminution of edemas [34], and bark extracts can inhibit membrane hemolysis. In addition, leaf extracts inhibit cyclooxygenase-2 (COX-2), one of the most important targets of anti-inflammatory drugs [35]. On the other hand, other researchers have focused on the anti-diabetic properties of *J. regia* in particular leaves, which showed excellent hypoglycemic effects [36,37].

To the best of our knowledge, this work is the first comparative study realized in kernels, leaves, husk, and bark of *J. regia* extracts based on the phytochemical composition and antioxidant, anti-inflammatory, and anti-diabetic activities. To date, no study reported the inhibitory effect of *J. regia* extracts on lipoxygenase (LOX).

In this work, we investigated the chemical screening of methanolic extracts of different *J. regia* parts: kernels (MWK), leaves (MWL), husk (MWH), and bark (MWB). In addition, enzymatic inhibitory activities of these part extracts were evaluated against enzymes involving inflammation and diabetes mellitus. Moreover, several methods were used to evaluate their antioxidant capacities (DPPH, FRAP, ABTS).

## 2. Materials and Methods

### 2.1. Chemicals

Methanol, Folin–Ciocalteau reagent, (7.5%) Na_2_CO_3_, gallic acid, (5%) sodium nitrite solution (NaNO_2_), 2,2′-azino-bis(3-ethylbenzothiazoline-6-sulfonic acid) (ABTS), 2,2-Diphenyl-1-picrylhydrazyl (DPPH), acetonitrile, (10%) aluminum trichloride (AlCl_3_), colchicine, Dimethyl sulfoxide (DMSO), ferric chloride (FeCl_3_), (0.1%) formic acid, nitrogen, iodine solution, PBS buffer, p-nitro-phenyl-α-D-glucopyranoside (p-NPG), (1%) Potassium ferricyanide, potassium persulfate, potassium phosphate buffer, quercetin, rutin, sodium hydroxide, sodium phosphate buffer, Kojic acid, (10%) trichloroacetic acid, Trolox, (4%) vanillin, hydrochloric acid (HCl) were analytical grade. L-Dopa, linoleic acid, tyrosinase, α-amylase, and α-glucosidase are purchased from Sigma.

### 2.2. Plant Material and Extraction

Leaves, husks, kernels, and bark of the plant *J. regia* were collected from the Taza region, Morocco. After the drying process, walnut parts were powdered and conserved in the dark at 4 °C. The extractions of leaves, husk, kernels, and bark powders (12.5 g) were realized by methanolic maceration (250 mL) for 24 h under agitation. Then, the mixture was filtered, the solvent was eliminated using a rotary evaporator at 60 °C, and the extracts were conserved at 4 °C.

### 2.3. Determination of Phenolic Contents

#### 2.3.1. Total Phenolic Content

The determination of total phenolic content in *J. regia* extracts was performed using the Folin–Ciocalteu procedure [38]. 0.5 mL of Folin–Ciocalteau reagent was added to 0.5 mL of each extract and agitated. Then, 4 mL of 7.5 % Na_2_CO_3_ (*w*/*v*) was added, and the reaction was incubated at 45 °C for 30 min. Calibration curves were established using gallic acid. The absorbance was measured at 765 nm, and the total phenolic contents were expressed as mg gallic acid equivalents per g of the dry weight of extract (mg GAE/g of extract).

#### 2.3.2. Total Flavonoid Content

The determination of the flavonoid content was carried out according to the aluminum trichloride method developed by Brighente et al. [39]. In test tubes, 1 mL of each of the extracts (1mg / mL) and 6.4 mL of distilled water were successively introduced, then 0.3 mL of the sodium nitrite solution (NaNO_2_ 5%) was added. After 5 min, 0.3 mL of aluminum trichloride (AlCl_3_ 10%) was added. After 6 min, 2 mL of sodium hydroxide (1 M) was added, and the solution was agitated and allowed to stand for 30 min. The absorbances were measured at 510 nm. Rutin was used as a standard under the same analytical conditions. The flavonoid content is expressed in mg of rutin equivalent per g of dried extract (mg RE/g of the extract).

#### 2.3.3. Total Tannin Content

The determination of total tannin contents was effectuated using the Julkunen–Tiitto [40] method. 50 μL of each extract was mixed with 1.5 mL of 4% vanillin, then 750 μL of hydrochloric acid HCl was added. After, the mixture was incubated for 20 min at room temperature in the dark. Colchicine was used as standard. The absorbances were measured at 500 nm. The results are expressed in milligrams equivalent to catechin per gram of the extract (mg CE/g of extract).

### 2.4. HPLC-DAD-ESI-MS/MS 

The chemical composition of *J. regia* methanolic extracts was determined by high-performance liquid chromatography (Hewlett-Packard 1100 Agilent Technologies) equipped with a DAD detector and an electrospray HP 1100 MSD API (Agilent-Technologies, Palo Alto, CA, USA) under analytical conditions reported by Pallaufa et al. [41]. A negative ionization mode, a capillary voltage of 3000 to 3500 V, and a fragmented variable of the order of 80 to 150 V were used. The column was a Poroshell 120 EC-C1, C18 (150 × 2.1) mm × 5 µm. The mobile phase was (A) 0.1% formic acid in the water, (B) acetonitrile. The established elution gradient was isocratic 15% B for 5 min, 15% B to 20% B over 5 min, 20–25% B over 10 min, 25–35% B over 10 min, 35–50% for 10 min. The total analysis time was 47 min, the flow rate was 0.5 mL/min. Double in-line detection was performed in the DAD using 280 nm and 370 nm as wavelengths and in a mass spectrometer (MS) connected to the HPLC system through the output of the DAD cell. MS detection was performed in a Qtrap API 3200 (Applied Biosystems, Darmstadt, Germany) equipped with an ESI source and a triple quadrupole ion trap mass analyzer. Zero-quality air was used as nebulizer gas (30 psi) and turbo gas for solvent drying (400 °C, 40 psi). Nitrogen served as a curtain (20 psi) and collision gas. The resolution of the quadrupoles was adjusted and the ion sputtering voltage was tuned at −4500 V (in negative mode). The MS detector has been set up to operate in two modes: Enhanced MS Analysis (EMS) and Enhanced Product Ion Analysis (EPIA) (EPI). To acquire an overview of all the ions in the sample, the EMS was utilized to capture the entire scan spectra. The defusing potential (DP) was −450 V, the input potential (EP) was −6 V, and the impact energy (CE) was −10 V. The spectra were recorded between *m*/*z* 100 and 1000 (in negative ion mode). For detected parent ion (s) discovered, the fragmentation pattern was then determined using EPI analysis under DP −50 V, EP −6 V, CE −25 V, and collision energy propagation (CES) 0 V [42].

### 2.5. Antioxidant Assay

#### 2.5.1. DPPH Method

A concentration of 1 mg/mL of each extract was prepared and diluted in methanol to obtain a range concentration from 10 to 1000 µg/mL. 1 mL of each sample concentration or standard was mixed with 0.5 mL of 0.2 mM DPPH methanolic solution. Trolox was used as a standard under the same conditions. Absorbances were measured at 517 nm after 30 min of incubation at dark conditions [43]. The radical scavenging ability (RSA) was expressed in % according to the following equation where the Abs control is the absorbance of the solution containing all reagents except sample (or standard). IC50 was calculated from the plot of RSA vs. extract concentration.

$$\mathrm{RSA}\%\;=\frac{\mathrm{Abs}\;\mathrm{control}\;-\mathrm{Abs}\;\mathrm{sample}}{\mathrm{Abs}\;\mathrm{control}}\times 100$$

#### 2.5.2. ABTS Method

The antioxidant activity was determined by the 2,2′-azino-bis(3-ethylbenzothiazoline-6-sulfonic acid) (ABTS) method [44]. 2 mM of ABTS was mixed with 70 mM of potassium persulfate. After incubation in the dark during 12–16 h, the ABTS^+^ solution was diluted with methanol to adjust absorbance to 0.700 ± 0.005 at 734 nm. Thus, 1 mL of each extract concentration (1 mg/mL) or standard was added to 2 mL of diluted ABTS solution to obtain a range concentration from 25 to 1000 µg/mL and incubated for 1 min, and the absorbance was measured at 734 nm. Trolox is used as a standard compound. Scavenging activity in this assay was expressed as the concentration of the extract required to inhibit 50% of the free radical scavenging activity.

#### 2.5.3. FRAP Method

The extract’s ferric ion-reducing power was determined by the FRAP method [45] with some modifications. A concentration of 1 mg/mL of each extract was prepared and diluted in methanol to obtain a range concentration from 5 to 50 µg/mL. Trolox was used as standard. 1 mL of each prepared extract or standard was mixed with 1.25 mL of 0.2 M sodium phosphate buffer (pH 6.6) and 1.25 mL of 1% potassium ferricyanide. The mixture was incubated at 50 °C for 20 min. After cooling, 1.25 mL of 10% trichloroacetic acid was added and centrifuged at 3000 rpm for 10 min. Finally, 1.25 mL of the supernatant was mixed with 1.25 mL distilled water and 0.25 mL FeCl_3_ solution (0.1%, *w*/*v*). Absorbances were measured at 700 nm, and results were expressed as the 50% effective concentration (EC_50_), which is the antioxidant concentration in mg/mL necessary to achieve an absorbance of 0.5.

### 2.6. Anti-Inflammatory Assay

#### 2.6.1. Lipooxidase Inhibition Assay

The lipooxidase inhibition assay was realized according to the procedure described by Debayo et al. [46]. A concentration of 1 mg/mL of each extract was prepared in methanol and diluted in 2M borate buffer to obtain a range concentration from 12.5 to 50 µg/mL. 12.5 µL of each extract concentration was added to 487.5 µL of 15-LOX (200 Units/mL) and kept at room temperature. After 5 min, 500 µL of linoleic acid dissolved in ethanol and diluted in the borate buffer were added to the enzymatic mixture and incubated for 5 min at room temperature. The absorbance was measured at 234 nm. Quercetin was used as a positive control, and DMSO was used as a negative control. The enzyme inhibition percentages were determined by the following equation.

$$\%\mathrm{Inhibition}=\frac{\left(\mathrm{Abs}\;\mathrm{extract}\;-\;\mathrm{Abs}\;\mathrm{blank}\right)}{\left(\mathrm{Abs}\;\mathrm{negative}\;\mathrm{control}\;-\;\mathrm{Abs}\;\mathrm{blank}\right)}\times 100\%$$

#### 2.6.2. Tyrosinase Inhibition Assay

The anti-tyrosinase activity was determined according to Huang et al. [47] procedure. A concentration of 1 mg/mL of each extract was prepared in methanol and diluted in 0.05 M PBS buffer (pH 6.5) to obtain a range concentration from 25 to 100 µg/mL. Tyrosinase enzymatic solution (333 U/mL) and L-Dopa (5 mM) were prepared in the PBS buffer. 50 µL of each extract concentration was mixed with 200 µL enzyme solution (3 U/mL), and the mixture was incubated at 37 °C. After 10 min, 500 µL of the substrate (L-Dopa) was added. The enzymatic reaction solution was then incubated for 30 min at 37 °C. The absorbance was measured at 510 nm. The percentage of inhibition was determined by the following formula, where Abs represents absorbance. Kojic acid was used as a standard.

$$\%\;\mathrm{of}\;\mathrm{inhibition}=\left(1-\frac{\;\left(\mathrm{Abs}\;\mathrm{control}\;-\;\mathrm{Abs}\;\mathrm{sample}\right)\;}{\mathrm{Abs}\;\mathrm{control}\;}\right)\times 100$$

### 2.7. Anti-Diabetic Activity

#### 2.7.1. α-Amylase Inhibition Assay

The effect of *J. regia* extracts on α-amylase activity was assessed according to Kusano et al. [48] method with some modifications. A concentration of 1 mg/mL of each extract was prepared in methanol and diluted in phosphate buffer (pH 6.9) to obtain a range concentration from 25 to 100 µg/mL. 200 µL of starch solution (substrate) was added to 100 µL of the buffer, and 250 µL of α-amylase (30 U/mL) was then incubated at 37 °C for 15 min. For the sample test, the enzyme was incubated with 250 µL of each extract concentration for 15 min. After adding substrate, the enzymatic reaction was conducted for 15 min and then stopped using 400 µL HCl (0.1 M). Total and remaining starch were measured at 630 nm after adding 500 µL of iodine solution (25 mM). For the positive control, acarbose was used. The percentage of inhibition was calculated by the following formula.

$$\%\;\mathrm{of}\;\mathrm{inhibition}=\left(1-\frac{\left(\mathrm{Abs}\;\mathrm{sub}\;-\mathrm{Abs}\;\mathrm{enz}+\mathrm{sub}\right)-\left(\mathrm{Abs}\;\mathrm{sample}-\;\mathrm{Abs}\;\mathrm{control}\right)\;}{\left(\mathrm{Abs}\;\mathrm{sub}-\mathrm{Abs}\;\mathrm{enz}+\mathrm{sub}\right)\;}\right)\times 100$$

#### 2.7.2. α-Glucosidase Inhibition Assay

The α-Glucosidase inhibition activity was tested according to Li et al. described method with slight modifications [49]. A concentration of 1 mg/mL of each extract was prepared in methanol and diluted in 1 M potassium phosphate buffer (pH 6.8) to obtain a range concentration from 250 to 1000 µg/mL. 100 µL of sample or acarbose (positive control), 380 µL of p-nitro-phenyl-α-D-glucopyranoside (p-NPG) (0.53 mM), and 250 µL of α-Glucosidase solution (0.015 Units/mL) were mixed in the buffer. After incubating at 37 °C for 20 min, 1mL of Na_2_CO_3_ (0.1 M) was added to quench the reaction. The IC_50_ value is determined by the concentration of α-Glucosidase inhibitor necessary to inhibit 50% of activity under assay conditions. The absorbance was measured at 405 nm, and the inhibition percentages were determined using the following equation:

$$\%\;\mathrm{of}\;\mathrm{inhibition}=\left(1-\frac{\;\left(\mathrm{Abs}\;\mathrm{enz}+\mathrm{sub}\;-\;\mathrm{Abs}\;\mathrm{sub}\right)-\left(\mathrm{Abs}\;\mathrm{sample}-\;\mathrm{Abs}\;\mathrm{control}\right)}{\left(\mathrm{Abs}\;\mathrm{enz}+\mathrm{sub}\;-\;\mathrm{Abs}\;\mathrm{sub}\right)\;}\right)\times 100$$

### 2.8. Statistical Analysis

Raw or log-transformed measured parameters were tested for normality and homogeneity of variance to meet the assumptions for parametric statistics. As the assumptions were violated for all parameters (extracts and assays), non-parametric analyses of variance, followed by a Dunn pairwise comparison test of means, were performed. The critical level of significance is set at 0.05.

## 3. Results and Discussion

### 3.1. Total Phenolic, Flavonoid, and Tocopherol Contents

The total phenolic content (TPC) analysis revealed a similar quantity of polyphenols in all methanolic extracts of *J. regia*, contrary to the total flavonoid content (TFC) analysis. The methanolic extract of bark and leaves showed the highest flavonoids quantity compared to husk and kernels, while the total tocopherol content (TTC) analysis showed the presence of tocopherols in low levels in bark methanolic extract (Table 1). 

### 3.2. HPLC-DAD-ESI-MS/MS Analysis

Qualitative analysis of *J. regia* L. methanolic extracts by HPLC-DAD-ESI-MS/MS was based on the retention times (Rt min), chemical formula, pseudo-molecular ions [MH], and MS/MS fragment ions (*m*/*z*).

In MWB, nine compounds were detected (Table 2). Kaempferol diglucoside was detected at [M − H]^−^ of *m*/*z* 447, as reported by Zeeshan et al. [50]. Three compounds were identified as Caffeic acid derivatives, among them caffeoyl hexoside (*m*/*z* 439) and caffeoyl hexose deoxyhexose (*m*/*z* 487), as reported by Appendino et al. [51].

In MWK, 14 phenolic compounds were identified (Table 3 belonging to various classes of natural compounds, notably fraxetin-8-O-glucoside (*m*/*z* 369), myricetin malonylglucoside (*m*/*z* 565), dicaffeoylquinic acid glycoside (*m*/*z* 677) as reported, respectively, in the studies [50,52,53]. Among the most dropping classes in this sample, the flavones such as Apigenin pentosyl glucoside with Rt 54.10 min and *m*/*z* 563 in addition to acacetin-7-O-rutinoside with *m*/*z* of 715 as reported by Gika et al. [54].

In the MWH extract, nine compounds were detected (Table 4). Several chemical classes have been identified as flavonols, notably Quercetin pentoside with [M−H]^−^of *m*/*z* 435 at 37.25 minas reported by Gika et al. [54]. Caffeic acid derivatives were detected by three *m*/*z* peaks 339, 427, and 487 at 11.64, 27.11, and 36.38 min, respectively, as reported by Appendino et al. [51] and Ieri et al. [52]. Moreover, the flavones were presented by acacetin-aglycone (*m*/*z* 283) and apigenin-7-O-glucoside (*m*/*z* 431).

In the MWL extract, 11 compounds were identified (Table 5). Several molecules belong to flavonols, notably myricetin malonylglucoside (*m*/*z* 565) and kaempferol-malonyl glucoside (*m*/*z* 533), as reported by Vagiri et al. (2012). The two compounds with [M−H]^−^ at *m*/*z* 437 and *m*/*z* 453 were identified as *p*-coumaroyl derivatives, as reported by Ieri et al. [52]. The carbohydrates were represented by Trigalloyl-glucose (*m*/*z* 633) and digalloyl-glucose (*m*/*z* 483), as reported by Gika et al. [54]. Thus, the caffeic acid derivatives were represented with [M−H]^−^ at *m*/*z* 531 and *m*/*z* 487.

*J. regia* extracts are rich in several bioactive components. The variation of phytochemical composition between *J. regia* parts may be due to the variation of patterns used in the synthesis, accumulation, and distribution of secondary metabolites, season, and environmental changes [55].

### 3.3. Antioxidant Activity: DPPH, FRAP, and ABTS Assays

**Figure 1:**
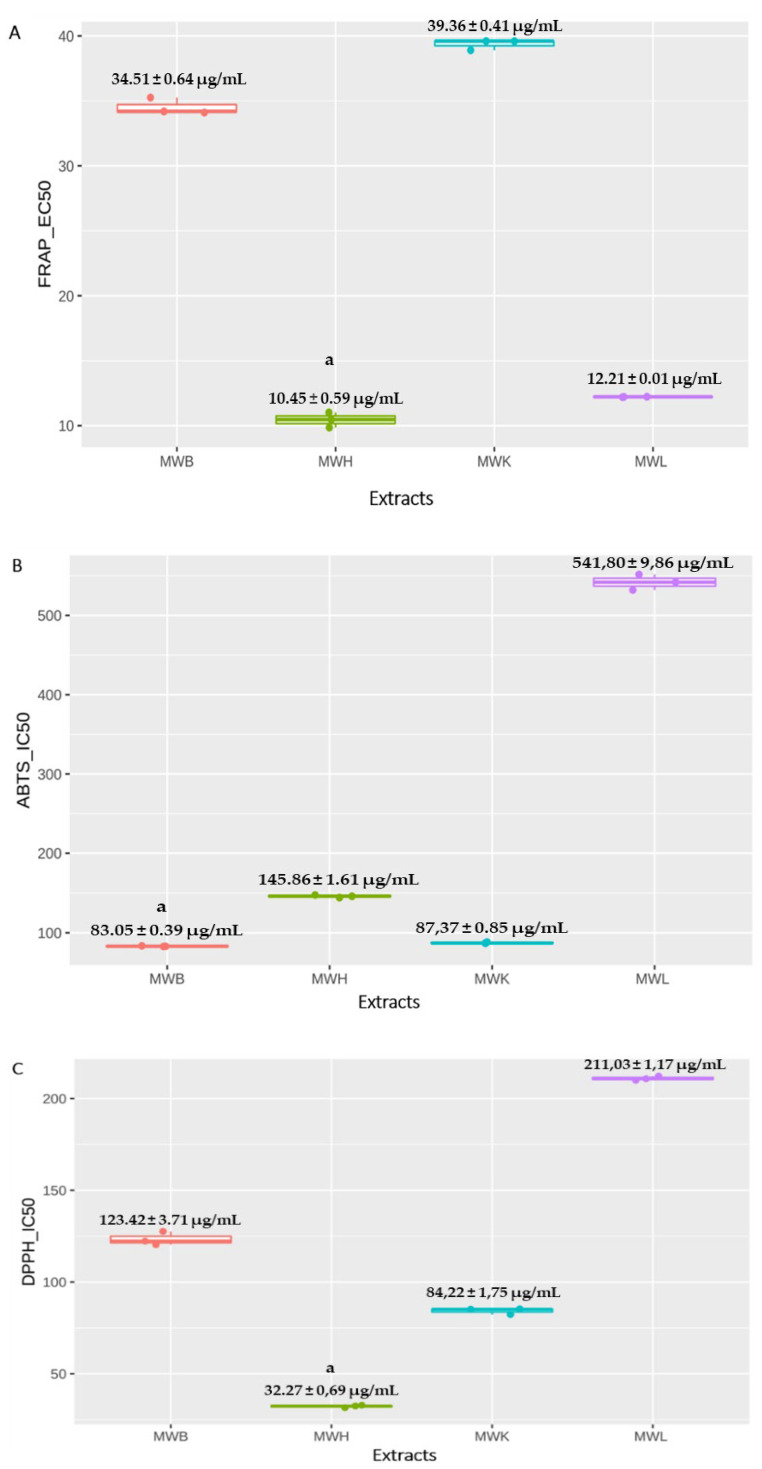
Boxplots showing FRAP (**A**), ABTS (**B**), and DPPH (**C**) radicals scavenging activities of *J. regia* methanolic extracts. ^a^ *p* < 0.05, Data expressed as mean ± SD. Abbreviations. MWB: Methanolic Extract of bark, MWH: Methanolic extract of Husk, MWK: Methanolic Extract of Kernels, MWL Methanolic Extract of Leaves.

The antioxidant activities of *J. regia* husk, bark, kernels, and leaves methanolic extracts were investigated using DPPH, FRAP, and ABTS methods. The results showed a concentration-dependent decrease in percentages of radicals. The data are reported as the concentration needed to inhibit 50% of radicals (IC_50_). Among all parts tested, the MWH extract showed the highest FRAP radicals’ neutralization capacity (*p* < 0.05), followed by MWL, MWB, and MWK extracts which were 10.45 ± 0.59 µg/mL, 12.21 ± 0.01 µg/mL, 34.51 ± 0.64 µg/mL and 39.36 ± 0.41 µg/mL (Figure 1a). However, Trolox used as standard control showed an EC_50_ of 5.25 µg/mL.

Contrary to FRAP assay results, the bark and the kernels methanolic extracts had the highest ABTS neutralization activity with IC_50_ less than 88 µg/mL (Figure 1b), while Trolox showed an important antioxidant capacity (IC_50_ = 15.2 µg/mL). In this study, the *J. regia* husk methanolic extract showed a pronounced ABTS neutralization activity (*p* < 0.05), which was higher than the results reported by Zhang et al. [30] with IC_50_ of 324.8 µg/mL vs. 145.86 ± 1.61µg/mL. Similarly, Bhatia et al. [56] investigated the ABTS neutralization activity of the methanolic extract of *J. regia* bark. They found a low neutralizing activity compared to our results with an IC_50_ of 601 µg/mL and 83.05 ± 0.39 µg/mL, respectively. On the other hand, the MWH extract had the strongest DPPH radical’s scavenging capacity with IC_50_ of 32.27 ± 0,69 µg/mL, which was significantly lower than that of leaves methanolic extract (*p* < 0.05) (Figure 1c). However, Trolox used as positive control inhibited DPPH radical at low concentrations (IC_50_ = 2.04). Ghasemi et al. [31] investigated the antioxidant activity of MWH extract obtained from 11 different regions in Iran. IC_50_ found in this previous study ranged from 122 ± 4.5 µg/mL to 302 ± 13 µg/mL, higher than IC_50_ obtained in our study. 

The methanolic extract of Tunisian *J. regia* bark showed a more pronounced radical scavenging activity (DPPH) compared to our results which were respectively IC_50_ of 36 µg/mL vs. 123.42 ± 3.71 µg/mL [57] contrary to the aqueous extract which showed a low antioxidant capacity with an IC_50_ of 582 µg/mL [56]. Concerning the methanolic extract of *J. regia* leaves, we obtained a similar result compared to the Jabli et al. study [58].

### 3.4. Anti-Inflammatory Activity

#### 3.4.1. Anti-Lipoxidase Activity

**Figure 2:**
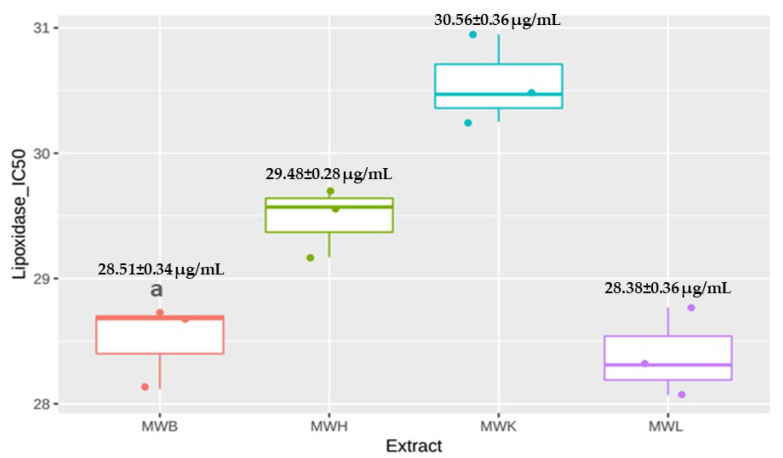
Effect of *J. regia* methanolic extracts on Lipoxygenase activity. ^a^ *p* < 0.05, Data expressed as mean ± SD. Abbreviations—MWB: Methanolic Extract of bark, MWH: Methanolic extract of Husk, MWK: Methanolic Extract of Kernels, MWL: Methanolic Extract of Leaves.

The anti-inflammatory activity of the methanolic extracts of *J. regia* was performed in vitro by lipoxygenase (LOX) inhibition activity. All methanolic extracts of *J. regia* leaves, bark, husk, and kernels exhibited high lipoxygenase activity inhibition, respectively, with IC_50_ of 28.38 ± 0.36, 28.51 ± 0.34, 29.48 ± 0.28, and 30.56 ± 0.36 µg/mL (Figure 2). These inhibitory activities are important compared to the standard (quercetin) (IC_50_ = 6.72 µg/mL). Lipoxygenases are a group of enzymes implicated in the inflammation process regulation, the production of inflammatory mediators, notably leukotrienes, by catalyzing the oxidation of poly-unsaturated fatty acids, essentially arachidonic acid and linoleic acid, in addition to catalyzing the formation of eicosanoids from arachidonic acid and immune response regulation [59]. The over-expression of lipoxygenase contributes to several pathologies, such as Alzheimer’s disease, diabetes, cancers, and cardiovascular disease, which further motivated research on natural compounds with anti-lipoxygenase activity [60]. 

Multiple studies have been interested in the anti-LOX activity of other plants. We found that *J. regia* kernels demonstrated an excellent LOX inhibition activity compared with the methanolic extracts of *Cydonia oblonga Mill* fruit and *Zanthoxylum armatum* fruit with IC_50_ of 99.30 µg/mL and 70.30 µg/mL, respectively [61,62]. Concerning the leaves’ methanolic extracts, some plants have a poor LOX inhibition activity, like *Cyclea barbata, Beilschmiedia penangiana, Veronica persica, Cassia alata*, and *Zanthoxylum armatum*. Others have a moderate anti-LOX activity compared with *J. regia*, notably *Artemisia nilagirica, Jatropha gossypifolia*, and *Ficus curtipes*, respectively, with IC_50_ of 128.20 µg/mL, 162.50 µg/mL, and 200.80 µg/mL. Others have similar LOX inhibition activity compared with *J. regia*, in particular *Memecylon malabaricum*, *Memecylon talbotianum*, and *Memecylon umbellatum*, respectively, with IC_50_ of 29.87 µg/mL, 34.60 µg/mL, and 39.19 µg/mL. The same for the bark methanolic extracts, *Pterocarpus erinaceus*, exhibited poor anti-LOX activity, while *Fraxinus rhynchophylla*, *Zanthoxylum armatum*, and *Beilschmiedia penangiana* had moderate LOX inhibition activity, respectively, with IC_50_ of 62.60 µg/mL, 90.50 µg/mL, and 176.80 µg/mL [63]. 

This activity is strongly related to the phytochemical profile of the plants. Flavonoids, as well as other plant secondary metabolites, are well-known inhibitors of pro-inflammatory mediators, the first and most well-known being benzoic acid. In this study, its derivatives were present with relatively high doses in *J. regia* methanolic leaves extract. 3,4-dihydroxybenzoic acid was investigated in numerous studies, and it was shown that it could directly bind the active site of soybean LOX, the latter sharing significant homologies with mammal’s LOX [64]. Other derivatives of benzoic acid are of significant interest to human and animal nutrition [65]. Quercetin also demonstrated in vitro inhibitory effects on lipoxygenases, particularly 5- and 15-lipoxygenases [66]. Kaempferol also exhibited anti-lipoxygenase-1 activity but with a lesser inhibitory effect compared to quercetin [67].

#### 3.4.2. Anti-Tyrosinase Activity

Tyrosinase is a metalloenzyme involved in the browning process of fruits and vegetables [68]. It also alters the melanogenesis process leading to chronic inflammation involved in several diseases. Melanin overproduction is associated with skin cancer and neurodegenerative disorders like Parkinson’s disease. According to the physiopathological role of tyrosinase, several research studies were conducted on tyrosinase inhibitory effects of natural compounds [4,69,70]. 

**Figure 3:**
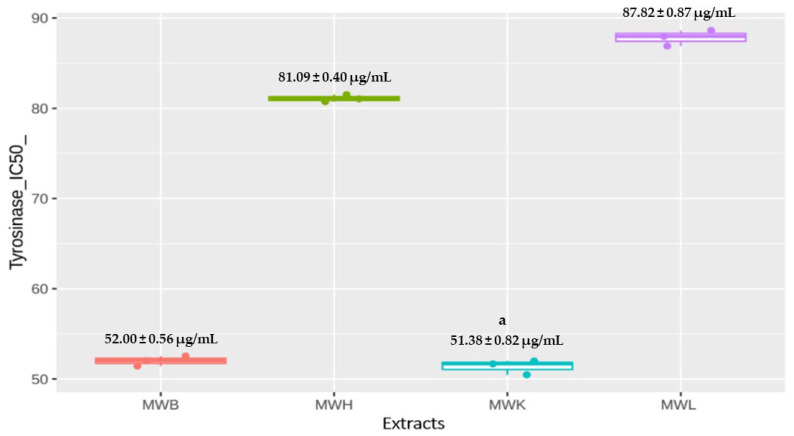
Effect of *J. regia* methanolic extracts on tyrosinase activity. ^a^ *p* < 0.05, Data expressed as mean ± SD. Abbreviations—MWB: Methanolic Extract of bark, MWH: Methanolic extract of Husk, MWK: Methanolic Extract of Kernels, MWL: Methanolic Extract of Leaves.

*J. regia* methanolic extracts exhibited strong inhibition of tyrosinase activity, especially the methanolic extracts of kernels and bark, respectively, with IC_50_ of 51.38 ± 0.82 µg/mL and 52.00 ± 0.56 µg/mL (*p* < 0.05) followed by the husk (IC_50_= 81.09 ± 0.40 µg/mL) and the leaves (IC_50_= 87.82 ± 0.87 µg/mL) (Figure 3). These results can be considered important since the used standard (kojic acid) showed an IC_50_ value equal to 5.28 µg/mL.

Previous studies on *J. regia* tyrosinase inhibitory activity reported that the water extract of *J. regia* leaves, seed, and husk have demonstrated an IC_50_ of 3.99 mg/mL, 8.83 mg/mL, and 10.13 mg/mL, respectively [71], the hydroethanolic extract of *J. regia* leaves presented an inhibition up to 50% at 751 ± 0.01 µg of extract/mL [72], in addition to a negligible antityrosinase activity in the extract of leaves collected in Turkey [73]. According to all these data, we note that the methanolic extract of *J. regia* collected from Morocco exhibits a significant anti-tyrosinase activity. Multiple studies have been focused on the anti-tyrosinase activity of other plant parts, notably the leaves of *Ceratonia siliqua*, the aerial parts of *Cleome arabica* and *Pituranthos scoparius*, whose exhibited moderate tyrosinase inhibition activity, respectively, with IC_50_ of 200 µg/mL, 124.4 ± 0.69 µg/mL and 125.01 ± 0.72 µg/mL. While other plant extracts had similar anti-tyrosinase activity to *J. regia* bark, such as *Harpephyllum caffrum* bark, contrariwise to the bark extracts of *Hyaenanche globosa* and *Cassipourea flanaganii*, which showed high activity against the tyrosinase with IC_50_ of 27.1 ± 042 µg/mL and 22.24 ± 1.32 µg/mL, respectively [74].

The methanolic extracts of kernels and bark phenolic profile showed mainly acacetin-aglycone and apigenin-7-O-glucoside. According to the literature, these flavones are encountered in many medicinal plants and are known for their therapeutic potential, including anti-inflammatory activities [75,76].

Our extracts showed the presence of myricetin, kaempferol, and quercetin which had been identified as tyrosinase inhibitors. Previous studies demonstrated that kaempferol and quercetin could inhibit the oxidation of L-DOPA catalyzed by tyrosinase [70]. Those molecules may be the actors behind the anti-tyrosinase activity of the methanolic extracts of *J. regia* parts.

### 3.5. Antidiabetic Activity

**Figure 4:**
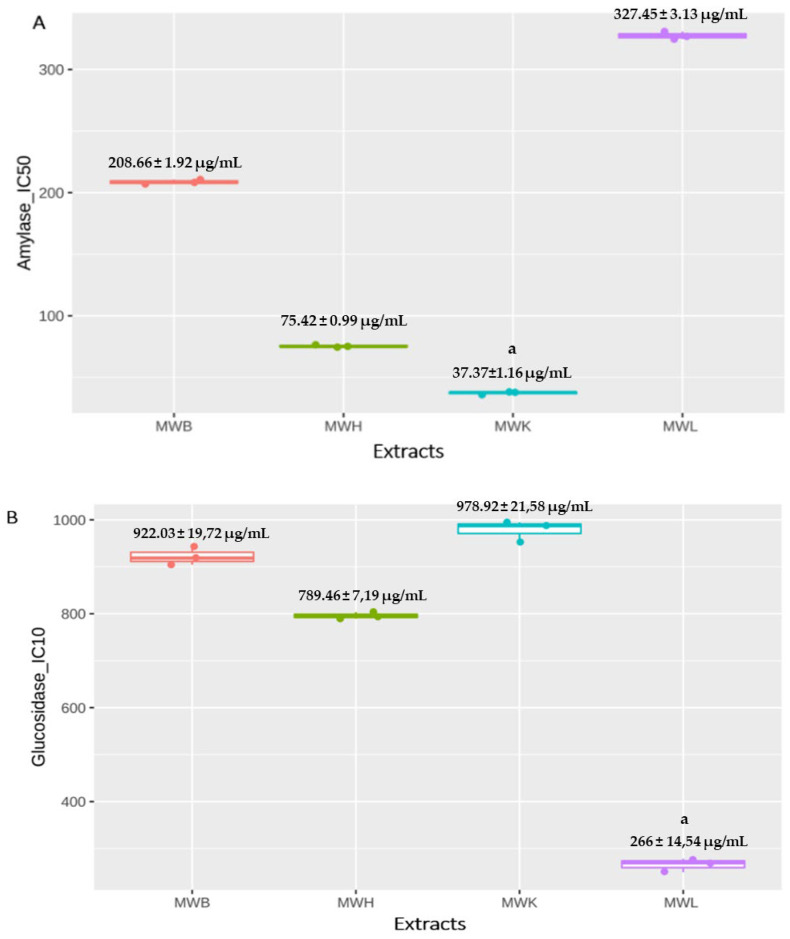
Effect of *J. regia* methanolic extracts on (**A**) α-amylase and on (**B**) α-glucosidase enzymatic activities. ^a^ *p* < 0.05, Data expressed as mean ± SD. Abbreviations—MWB: Methanolic Extract of bark, MWH: Methanolic extract of Husk, MWK: Methanolic Extract of Kernels, MWL: Methanolic Extract of Leaves.

The antidiabetic activity of the different extracts of *J. regia* was assessed using the amylase and glycosidase inhibition assays. MWK and MWH highly inhibit the amylase activity with IC_50_ values equal to 37.37 ± 1.16 µg/mL and 75.42 ± 0.99 µg/mL, respectively, which are significantly higher (*p* < 0.05) compared to MWL (IC_50_ = 327.45 ± 3.13 µg/mL) (Figure 4a). Certainly, *J. regia* methanolic leaves extract (MWL) has the weakest inhibition effect on the α-amylase activity of all other extracts (*p* = 0.07), but it remained more effective compared with *J. regia* aqueous extract, which demonstrated an inhibition of 60% of amylase with 0.4 mg/mL [77]. These results are not significantly important compared with the inhibitory value of α-amylase shown by acarbose (26.11 μg/mL). Likewise, MWB extract released 50% of amylase activity at a concentration less than 210 μg/mL, and this remains more effective compared with bark aqueous extract of *J. regia* collected in Oued Amlil located in Taza region that showed an IC_50_ of 5445.33 ± 82.58 μg/mL [78].

Quantitative analysis of *J. regia* extracts showed that the methanolic kernels extract has more polyphenols than flavonoids, and this may explain its effect on amylase because it has been shown that polyphenols bind with this digestive enzyme affecting starch hydrolysis activity in the small intestine reducing the absorption of glucose and consequently improving glycemic status in patients with diabetes [79,80]. This effect is mediated mainly by condensed and hydrolyzable tannins [79], and indeed, the qualitative analysis by HPLC-DAD-ESI-MS/MS demonstrated the dominance of gallotannin in this extract. In addition to that, the HPLC-DAD-ESI-MS/MS analysis showed that kernels of methanolic extract of *J. regia* contain a caffeoylquinic acid derivative, including dicaffeoyl-quinic acid glycoside. It was shown that this polyphenol class contributes as the most active principle against diabetes in vitro, and that may explain the excellent effect of this plant extract.

The evaluation of the inhibitory activity of *J. regia* extracts on α-glycosidase showed moderate results (compared to acarbose IC_50_ = 0.35 μg/mL). MWL has the lowest IC_10_ (266 ± 14.54 μg/mL) compared to MWB (922.03 ± 19.72 μg/mL), MWH (789.46 ± 7.19 μg/mL), MWK (978.92 ± 21,58 μg/mL) (Figure 4b). This effect may be due to caffeoylquinic acids, which can strongly inhibit glucosidase [81]. Previous studies had investigated the anti-glycosidase activity of other plants, notably the leaves of *Annona senegalensis* and *Liquidambar formosana*, which exhibited high glycosidase inhibition activity, respectively, with IC_50_ of 97 μg/mL and 59 μg/mL compared to *J. regia* methanolic extracts [82]. *J. regia* leaf extracts have previously demonstrated an effective effect on diabetes in vitro and also in vivo by glycemia levels normalization via the inhibition of glucose-6-phosphate translocase and transporter GLUT2 in addition to the reduction of cholesterol synthesis by hydroxymethyl glutaryl-CoA reductase inhibition on rats thanks to caffeoylquinic acid and quercetin [35,37,83] and even in humans [84].

### 3.6. Correlations among J. regia Biochemical Activities in Different Extracts

**Figure 5:**
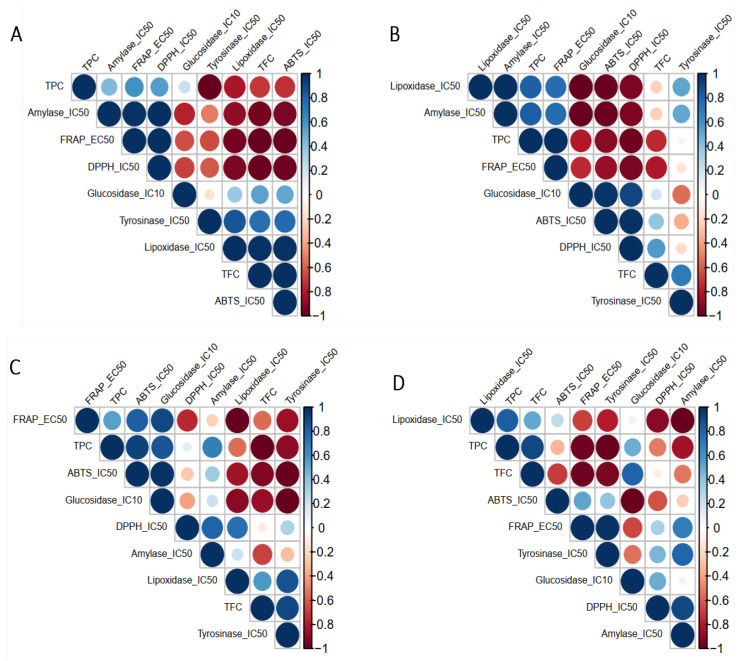
Correlations among chemical activities (antioxidative, anti-inflammatory, antidiabetic, and anti-tyrosinase) within each *J. regia* extract (**A**) Methanolic Extract of bark, (**B**) Methanolic extract of Husk, (**C**) Methanolic Extract of Kernels, and (**D**) Methanolic Extract of Leaves.

To better understand the potential activities of different *J. regia* part extracts, we investigated the correlations among the biochemical activities, notably antioxidative activity, anti-inflammatory activity, and antidiabetic activity within each *J. regia* extract (Figure 5). The analysis showed positive correlations between the anti-amylase activity and the antioxidant in the four extracts represented by the FRAP and DPPH assays. On the other hand, the anti-glucosidase activity of MWH and MWK was also positively correlated with the antioxidant activity, while this activity was correlated with TFC and TPC in MWL.

**Figure 6:**
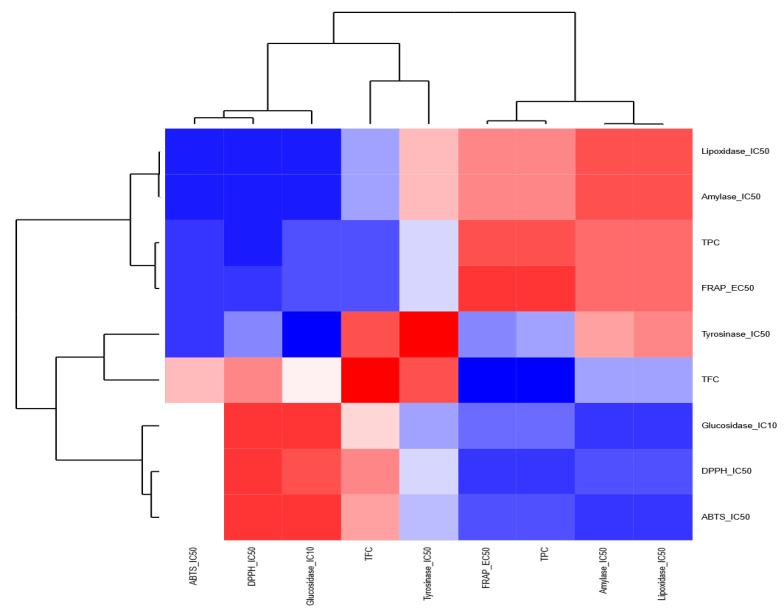
Heatmap highlighting the relationships among the chemical screenings within the methanolic extract of bark.

**Figure 7:**
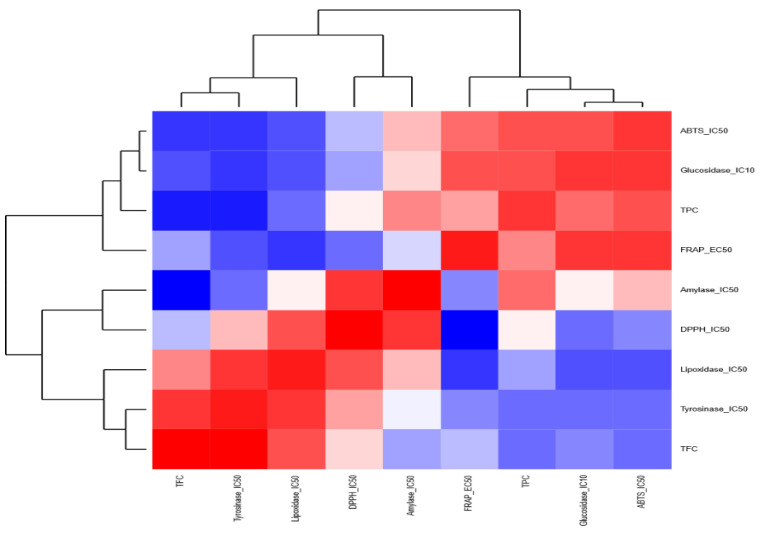
Heatmap highlighting the relationships among the chemical screenings within the methanolic extract of kernels.

As for the anti-inflammatory properties, the anti-LOX activity was correlated with the TFC and the antioxidant activity in MWB, MWH, and MWK, contrary to MWL, the anti-LOX activity was correlated with TPC and TFC. The anti-tyrosinase activity of MWK and MWL was correlated positively with TFC and FRAP, respectively. When we used a heatmap coupled with a dendrogram to highlight the relationships among different chemical screening within each extract, we found out that for the MWB extract (Figure 6), the chemical screenings are clustered into two distinct groups: (i) the first group contained FRAP, TPC, amylase et lipoxidase and (ii) the second group harbored tyrosinase, TFC, glycosidase, DPPH, and ATBS. Similarly, for the MWK (Figure 7) extract, the heatmap showed two groups: (i) the first one included TFC, ABTS, lipoxidase, tyrosinase, and glycosidase; (ii) the second one contained TPC, DPPH, FRAP, and amylase.

**Figure 8:**
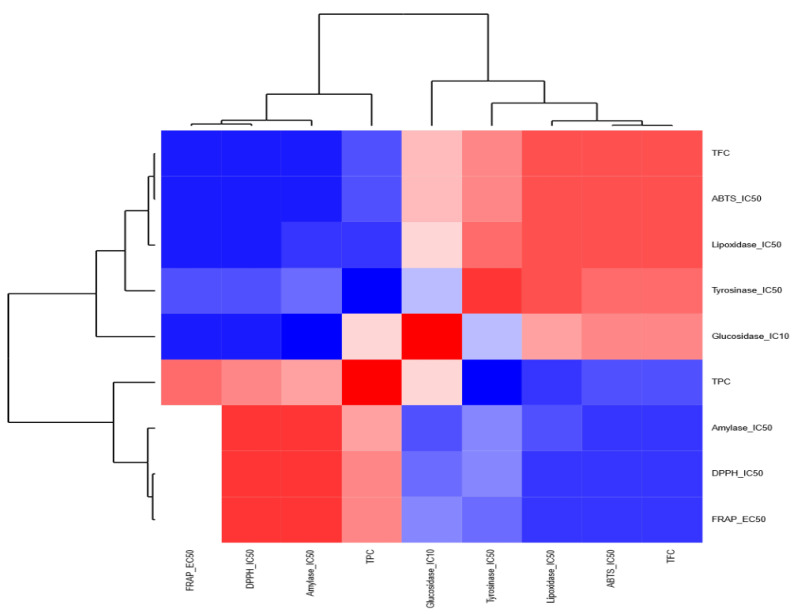
Heatmap highlighting the relationships among the chemical screenings within the methanolic extract of husk.

**Figure 9:**
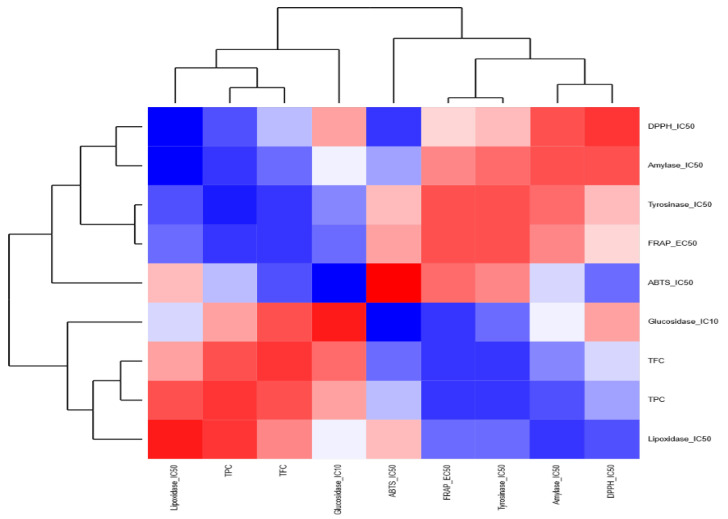
Heatmap highlighting the relationships among the chemical screenings within the methanolic extract of leaves.

In the case of the MWH extract (Figure 8), (i) the first group is composed of ABTS, TPC, FRAP, and glycosidase, and (ii) the second group comprises amylase, lipoxidase, tyrosinase, TFC, and DPPH. For the MWL extract (Figure 9), there are two groups: (i) the first group encompasses ABTS, FRAP, amylase, tyrosinase, and DPPH, and (ii) the second group consists of glycosidase, lipoxidase, TPC, and TFC.

Definitively the chemical activity profiles varied deeply among the *J. regia* extracts; this finding should be taken into account when using the different extracts for therapeutic purposes.

## 4. Conclusions

*J. regia* is a well-known species widely used for its nutritional benefits and also for its therapeutic properties, as reported in numerous ethnobotanical studies conducted in many countries.

This study is the first to emphasize the variability in antioxidant and biological characteristics across the various parts of the walnut. This variability will be used to guide in vivo studies and, consequently, the uses by herbalists of those parts of plants exhibiting the most relevant activities.

Results highlighted the potential antidiabetic properties of kernels and husk extracts as well as the anti-inflammatory properties of bark extract. In fact, the phenolic profile determined by HPLC-DAD-ESI-MS/MS showed the richness of *J. regia* in bioactive compounds. However, further investigations concerning the isolation of main chemical compounds, as well as the evaluation of their antioxidant, antidiabetic, and anti-inflammatory effects, are required to determine the molecular mechanisms involved in these biological activities. Moreover, in vivo explorations and toxicological investigations are needed to determine the main pharmaceutical parameters of these compounds as well as to validate their safety.

## Figures and Tables

**Table 1:** Total penolic, flavonoid, and tocopherol contents of *Juglans regia* methanolic extracts. ^a^ *p* < 0.05, Data expressed as mean ± SD. Abbreviations; GA: Gallic acid; RU: Rutin; Cat: Catechin, MWB: Methanolic Extract of bark, MWH: Methanolic extract of Husk, MWK: Methanolic Extract of Kernels, MWL Methanolic Extract of Leaves.

	TPC	TFC	TTC
	EqGA mg/g	EqRU mg/g	EqCat mg/g
MWL	389.40 ± 2.74	306.36 ± 9.73	–
MWH	306.36 ± 4.74	66.07 ± 2.68	–
MWK	406.95 ± 7.60	18.44 ± 4.75 ^a^	–
MWB	413.71 ± 9.73	395.71 ± 39.44	5.44 ± 1.07

**Table 2:** Identification of main phenolic compounds of *J. regia* bark extracts using HPLC-DAD-ESI-MS/MS.

No	Rt (min)	MS/MS (*m*/*z*)	Fragment	Compounds	Chemical Class
1	4.63	439	169/183/341	Caffeoyl Hexoside	Caffeic acid derivatives
2	10.47	435	177/183/195/331/435	Quercetin Pentoside	Flavonols
3	17.85	369	183/195/233/331/369	Fraxetin-8-O-glucoside	Hydroxycoumarin
4	18.74	615	233/255/331/447/615	Quercetin Galloyl-glucoside	Flavonols
5	19.61	545	233/255/435/447/545	O-methyl-epicatechin-sulfate-O-glucoside	Flavanols
6	20.30	447	299/447	Kaempferol Glucoside	Flavonols
7	21.81	331	285/331	Gallic Acid glucoside	Phenolic acid
8	32.26	487	375/457/487	Caffeoyl hexose deoxyhexose	Caffeic acid derivatives
9	37.32	893	297/397/527	Di-caffeic acid derivatives	Caffeic acid derivatives

**Table 3:** Identification of main phenolic compounds of *J. regia* kernel extracts using HPLC-DAD-ESI-MS/MS.

No	Rt (min)	MS/MS (*m*/*z*)	Fragment	Compounds	Chemical Class
1	4.65	369	269/293/315/369	Fraxetin-8-O-glucoside	Hydroxycoumarin
2	10.68	565	255/345/483/565	Myricetin malonyl-glucoside	Flavonols
3	11.50	665	113/335/407/665	Gallotannin	Phenolic acid
4	20.11	691	195/435/545/691	Quercetin galloyl-glycoside	Flavonols
5	27.94	593	117/253/425/593	Kaempferol glucosyl-rhamnoside	Flavonols
6	32.20	563	239/365/427/563	Apigenin pentosyl glucoside	Flavones
7	37.25	737	311/409/435/737	Quercetin pentoside	Flavonols
8	42.21	445	291/309/345/445	Apigenin-O-glucuronide	Flavones
9	43.25	701	311/409/663/701	Tiliroside	Flavonols
10	50.90	677	295/313/452/677	Dicaffeoylquinic acid glycoside	Phenolic
11	51.38	505	141/387/411/505	Quercetin Malonyl Hexoside 1	Flavonols
12	54.10	563	255/281/563	Apigenin Peptosyl glucoside	Flavones
13	54.66	715	141/279/309/715	Acacetin-7-O-rutinoside	Flavone
14	58.04	903	283/667/751/903	Tetragalloyl glucose	Glucide

**Table 4:** Identification of phenolic compounds of *J. regia* husk extracts using HPLC-DAD-ESI-MS/MS.

No	Rt (min)	MS/MS (*m*/*z*)	Fragment	Compounds	Chemical Class
1	4.72	197	147/183/197	Dihydroxybenzoic acid derivative	Phenolic acid
2	10.20	283	255/283	Acacetin aglycone	Flavone
3	11.64	339	177/197/339	Caffeoyl-D-glucose	Caffeic acid derivatives
4	17.41	507	191/207/435/507	Quercetin O hexoside 1	Flavonols
5	18.97	431	207/385/417/431	Apigenin-7-O-glucoside	Flavones
6	27.11	427	297/339/373/427	Caffeoyl derivative	Caffeic acid derivatives
7	32.20	529	141/293/313/441/529	p-Coumaroyl derivative	Coumaric acid
8	36.38	487	157/171/267/409/433/487	Caffeoyl hexose-deoxyhexoside	Caffeic acid derivatives
9	37.25	435	359/409/417/435	Quercetin pentoside	Flavonols

**Table 5:** Identification of phenolic compounds of *J. regia* leaves extracts using HPLC-DAD-ESI-MS/MS.

No	Rt (min)	MS/MS (*m*/*z*)	Fragment	Compounds	Chemical Class
1	4.28	437	145/171/249/437	*p*-Coumaroyl derivative	Coumaric acid
2	11.60	453	159/177/339/383/437/453	*p*-Coumaroyl derivative	Coumaric acid
3	16.65	531	317/ 417/ 447 /483/531	Caffeoyl derivative	Caffeic acid derivatives
4	18.80	533	447/ 463/515/533	Kaempferol-malonylglucoside	Flavonols
5	19.92	565	395/435/533/565	Myricetin malonyl-glucoside	Flavonols
6	20.47	631	171/263/459/533/631	Quercetin galloyl-glycoside	Flavonols
7	20.93	583	183/201/485/583	Myricetin acetylglycoside	Flavonols
8	21.61	711	183/255/389/485/711	Quercetin-7-O-hexoside-3-O- (malonyl) hexoside	Flavonols
9	22.04	633	325/469/595/633	Trigalloylglucose	Glucide
10	32.28	483	313/339/483	Digalloylglucose	Glucide
11	36.43	487	313/343/355/487	Caffeoyl hexose-deoxyhexoside	Caffeic acid derivatives

## Data Availability

Not applicable.

## Abbreviations

ABTS	2,2′-azino-bis(3-ethylbenzothiazoline-6-sulfonic acid)
Cat	Catechin
CE	Impact Energy
CES	Collision Energy Propagation
DP	Defusing Potential
DPPH	2,2-Diphenyl-1-picrylhydrazyl
EMS	Enhanced MS Analysis
EP	Input Potential
EPIA	Enhanced Product Ion Analysis
FRAP	Ferric Reducing Antioxidant Power
GA	Gallic acid
GLUT2	Glucose transporter 2
HCl	Hydrochloric acid
HPLC	High-Performance Liquid Chromatography
*J. regia*	*Juglans regia*
LOX	Lipoxygenase
MS	Mass Spectrometer
MWB	Methanolic Extract of Bark
MWH	Methanolic extract of Husk
MWK	Methanolic Extract of Kernels
MWL	Methanolic Extract of Leaves
ROS	Reactive Oxygen Species
RU	Rutin
TE	Trolox Equivalent
TFC	Total Flavonoid Content
TPC	Total Phenolic Content
TTC	Total Tocopherol Content
